# Enhancing Dementia Care Via GUIDE: Focus on Older Adults Living Alone With Limited Supports

**DOI:** 10.1093/geroni/igaf122.4236

**Published:** 2025-12-31

**Authors:** Elena Portacolone, Krista Harrison, Jarmin Yeh, Sarah Dulaney, Katherine Possin, Matthew Growdon, Andrew Cohen, Robyn Stone

**Affiliations:** University of California, San Francisco, San Francisco, California, United States; University of California, San Francisco, San Francisco, California, United States; University of California, San Francisco, San Francisco, California, United States; University of California, San Francisco, San Francisco, California, United States; University of California, San Francisco, San Francisco, California, United States; University of California, San Francisco, San Francisco, California, United States; Yale School of Medicine, New Haven, Connecticut, United States; LeadingAge, Washington, District of Columbia, United States

## Abstract

**Background:**

Approximately one-fourth of older adults with dementia live alone in the United States (about 1.7 million people), often with limited or no caregiver support, making them a particularly challenging population to care for healthcare providers. These individuals often have limited support from caregivers, and it is unclear whether they will benefit from the Guiding an Improved Dementia Experience (GUIDE) national demonstration model, launched by the Centers for Medicare and Medicaid Services in July 2024, which emphasizes support for patient/caregiver dyads. To address this knowledge gap, we elicited the perspectives of national dementia experts on the GUIDE model’s ability to meet the needs of older adults living alone with limited caregiver support.

**Methods:**

From October-November 2024, teleconference interviews were conducted with 10 national dementia policy experts working in research, consulting, and advocacy institutions. Interview transcripts were analyzed using a combined inductive and deductive content analysis approach.

**Results:**

All experts concurred that obstacles may prevent older adults living alone with dementia from benefitting fully from GUIDE, for 5 reasons: : 1) no benefit exists equivalent to what is provided for caregivers’ respite ($2,500/year); 2) monthly reimbursements of $215 may be insufficient to support this high-needs population; 3) GUIDE-related medication plans may be impractical; 4) navigators are mostly available via telephone rather than in-home; 5) the GUIDE evaluation ignores living arrangements and includes only interviews with caregiver/patient dyads.

**Conclusions:**

Findings underscore the importance of developing comprehensive care interventions to effectively support older adults with dementia who live alone with limited caregiver support.

